# Sequence Complexity Effects on Speech Production in Healthy Speakers and Speakers with Hypokinetic or Ataxic Dysarthria

**DOI:** 10.1371/journal.pone.0077450

**Published:** 2013-10-16

**Authors:** Kevin J. Reilly, Kristie A. Spencer

**Affiliations:** 1 Department of Speech-Language Pathology & Audiology, Northeastern University, Boston, Massachusetts, United States of America; 2 Department of Speech & Hearing Sciences, University of Washington, Seattle, Washington, United States of America; Utrecht University, Netherlands

## Abstract

The present study investigated the effects of sequence complexity, defined in terms of phonemic similarity and phonotoactic probability, on the timing and accuracy of serial ordering for speech production in healthy speakers and speakers with either hypokinetic or ataxic dysarthria. Sequences were comprised of strings of consonant-vowel (CV) syllables with each syllable containing the same vowel, /a/, paired with a different consonant. High complexity sequences contained phonemically similar consonants, and sounds and syllables that had low phonotactic probabilities; low complexity sequences contained phonemically dissimilar consonants and high probability sounds and syllables. Sequence complexity effects were evaluated by analyzing speech error rates and within-syllable vowel and pause durations. This analysis revealed that speech error rates were significantly higher and speech duration measures were significantly longer during production of high complexity sequences than during production of low complexity sequences. Although speakers with dysarthria produced longer overall speech durations than healthy speakers, the effects of sequence complexity on error rates and speech durations were comparable across all groups. These findings indicate that the duration and accuracy of processes for selecting items in a speech sequence is influenced by their phonemic similarity and/or phonotactic probability. Moreover, this robust complexity effect is present even in speakers with damage to subcortical circuits involved in serial control for speech.

## Introduction

Since Lashley’s seminal paper [1] outlining the “problem of serial order” in speech production and other movement behaviors, the process underlying fluent ordering of speech sounds and syllables has been an area of active research interest. Contemporary accounts of serial control for speech are based largely on analyses of speech error rates and reaction times in different experimental conditions. These datasets provide compelling support for the idea that all or most items in an upcoming speech sequence are ‘activated’, or available for selection, in a response buffer(s) prior to initiation of the sequence [1–4]. The order of sequence items results from competitive processes that organize items into larger frames and select the next item, or group of items, to be produced [5–10]. The emphasis on chronometric measures of speech reaction time has provided detailed accounts of the factors that influence the encoding and activation of sequences in a response buffer. However, the lack of corresponding data on within-sequence chronometric measures, such as segment and syllable durations, raises questions about the effects of these factors on the selection of individual speech items and how this selection unfolds over the course of sequence production. The current study evaluated the effects of sequence complexity on the production of speech sequences in healthy speakers, speakers with hypokinetic dysarthria secondary to Parkinson’s disease, and speakers with ataxic dysarthria. An analysis of healthy speakers’ production of speech sequences assessed the effects of sequence complexity on the time course for selecting speech items throughout sequence production. A comparison of these findings with those of speakers with hypokinetic dysarthria and speakers with ataxic dysarthria evaluated whether serial processing of sequence complexity is affected in these clinical populations. 

Analyses of speech errors and chronometric measures provide benchmark findings for models of speech serial control. These behavioral methods have helped to identify contextual factors, such as sequence length and practice, that systematically affect errors and reaction times [3,4,11,12]. There is considerable evidence that the processes responsible for fluent speech sequencing are also sensitive to sequence complexity, which in the present study reflected the properties of individual sequence items and their relation to other items in the sequence. Relevant metrics for characterizing this type of complexity include similarity-based metrics, which describe the similarity of sequence items to each other, and usage-based metrics which describe the frequency with which items or combinations of items in a sequence occur in a language. For example, numerous studies have reported that the similarity of to-be-produced speech items has systematic effects on speech reaction times and error rates [2,10,13–17]. Similarity can be achieved through manipulation of the phonologic or phonemic aspects of a speech sequence. Phonological similarity is accomplished when the syllables or words in the sequence contain the same phoneme(s) in particular syllable- or word-positions. The present study investigated similarity effects at the phonemic level, which refers to the similarity among phonemes in a sequence and is characterized by the number of phonetic features these phonemes share. Phonemic similarity effects were demonstrated by Meyer and Gordon [13] who found that speakers exhibited longer speech reaction times and higher error rates during a response-priming task when mismatched prime-target trials shared phonetic features compared to mismatched trials that did not share phonetic features. Similar findings were reported by Rogers and Storkel [14] who investigated phonemic similarity effects in 84 speakers who read individual words as rapidly as possible following their presentation on a computer monitor. In this study, the same word was presented between three and eight times in a row before a novel word was presented. The investigators observed longer speech reaction times and higher error rates when the novel word shared phonetic features with the repeated word compared to when the novel and repeated words did not. Phonemic similarity effects on speech error rates have also been documented during speeded production of word pairs [18], production of tongue twisters [19,20] and immediate serial recall [21–23]. In addition, spontaneous sound exchange errors are significantly more likely to occur when the exchanged sounds share phonetic features than when they do not [2]. Together, the interference effects associated with production of phonemically similar sequences indicate that this factor influences the complexity of speech sequences.

Another factor that influences sequence complexity is the phonotactic probabilities of sounds and syllables in a sequence. Phonotactic probability describes the frequency of occurrence of phonemes, syllables, and phoneme combinations in a language or at a particular location in a word. For example, faster speech reaction times and lower error rates have been observed during production of word and non-word stimuli containing high vs. low frequency syllables [24–26] as well as high vs. low frequency sound sequences [27,28]. In addition, speech error analyses have found that speech errors tend to preserve phonotactic constraints such that a sound segment is significantly more likely to be erroneously inserted into a syllable position where it occurs more frequently than into one where it occurs less frequently [29–31]. These data indicate that, like phonemic similarity, interference effects associated with phonotactic probability contribute to the complexity of a sequence. 

Despite advances in understanding serial control in healthy speakers, there are few studies addressing speech sequencing in speakers with dysarthria. Neuroimaging studies of speech sequence production indicate that serial control for speech is distributed over multiple regions of cortex as well as subcortical areas in the basal ganglia and cerebellum [8,32–37]. The distributed nature of serial control for speech raises the possibility that speakers with dysarthria secondary to central nervous system lesions may exhibit deficits in the sequencing of sounds and syllables for speech production. In particular, reports of sequencing deficits in speakers with damage to either the cerebellum [38–40] or the basal ganglia [41–44] indicate that damage to either of these structures may impact serial processing for speech. In addition, many contemporary accounts of cerebellar and basal ganglia function highlight the importance of these structures to the serial control of both speech [8,45–48] and non-speech movements [49–52]. Thus, sequence complexity effects on speech error rates and segment and syllable durations were also evaluated in speakers with hypokinetic dysarthria secondary to Parkinson’s disease and speakers with ataxic dysarthria to determine whether these speaker groups exhibited deficits in serial performance related to either similarity-based or usage-based aspects of serial processing for speech production. 

In summary, research findings regarding speech error rates and reaction times indicate that the complexity of serial processing for speech is influenced by the phonemic similarity and phonotactic probability of sequence items. To date, there are few studies that address how these factors influence the selection of speech items over the course of sequence production and almost no studies addressing these processes in speakers with dysarthria. To examine this issue, the current study evaluated measures of sound and syllable durations during production of sequences that varied in complexity based on their phonemic similarity and phonotactic probability. Data for the current study consisted of speech sequence productions from Experiment 2 of the Spencer and Rogers [53] reaction time study. In that study, the authors observed longer reaction times during high versus low complexity sequences. The present analyses of sound and syllable durations were designed to supplement the reaction time finding of Spencer and Rogers [53] and provide an integrated account of complexity effects both prior to and during sequence production. 

## Methods

### Participants

The present study was approved by the Institutional Review Board of the University of Washington. All participants gave their written informed consent. Speakers were ten adults with hypokinetic dysarthria (mean age = 64.1, SD = 14.2), five adults with ataxic dysarthria (mean age = 30.0, SD = 6.2), and fifteen healthy control speakers (mean age = 51.0, SD = 19.3) with no reported history of speech, language, or neurological impairment [53]. Neurologic diagnosis was confirmed via neurology reports obtained from medical records. Speakers with hypokinetic dysarthria were evaluated under optimum medication per self-report. Information regarding the speech and non-speech clinical characteristics is displayed in Table 1 for the speakers with hypokinetic dysarthria and in Table 2 for speakers with ataxic dysarthria. All of the speakers with hypokinetic dysarthria had diagnoses of Parkinson’s disease except for speaker H1, who was diagnosed with multiple system atrophy (MSA), and speaker H3, who was diagnosed with corticobasal degeneration (CBD). The complexity of basal ganglia circuits and connectivity [54] raises the possibility that sequencing deficits are not uniform across basal ganglia pathologies but are specific to the type and location of the pathology. For this reason, statistical analyses of within- and between-group measures of serial performance were limited to speakers within the hypokinetic group who had a diagnosis of Parkinson’s disease. Qualitative comparisons between the speakers with Parkinson’s disease and the two speakers with hypokinetic dysarthria from MSA or CBD provided an informal measure regarding the specificity of serial performance characteristics in speakers with different pathologies of the basal ganglia. In addition, one healthy speaker (S002) was diagnosed with Amyotrophic Lateral Sclerosis approximately a year after participating in the Spencer and Rogers [53] study and that speaker’s data were excluded from the present study. 

**Table 1:** Clinical characteristics of participants with hypokinetic dysarthria.

Speaker	Age	Sex	Diagnosis	Duration	Tremor	Gait	Akinesia/ Bradykinesia	Dysarthria-Dysphonia	Sentence Intelligibility*
H1	66	F	MSA	6 years	–	++	+	moderate	95%
H2	78	F	PD	3 years	–	+	+	mild	100%
H3	35	F	CBD	7 years	–	+++	+++	moderate-severe	99%
H4	67	M	PD	2 years	+	+	+	mild	98%
H5	60	F	PD	4 years	+	+	++	mild	99%
H6	58	M	PD	2 years	+	–	–	moderate	96%
H7	77	F	PD	2 years	+	–	–	mild	99%
H8	65	F	PD	16 years	+	+	(dyskinesias)	mild	99%
H9	84	M	PD	5 years	++	+	+	moderate	94%
H10	51	F	PD	11 years	–	+	(dyskinesias)	negligible	100%

MSA, multi-system atrophy; PD, Parkinson’s disease; CBD, corticobasal degeneration; – no impairment; + mild impairment; ++, moderate impairment; +++ severe impairment.

* Sentence intelligibility was determined by the Assessment of Intelligibility of Dysarthria Speech [77].

**Table 2:** Clinical characteristics of participants with ataxic dysarthria.

Speaker	Age	Sex	Diagnosis	Duration	Upper limb signs	Gait signs	Oculomotor signs	Dysarthria Severity	Sentence Intelligibility
A1	38	F	Cerebellar toxicity	10 years	–	+	–	Moderate	98%
A2	22	F	Friedreich's Ataxia	8 years	+	+++	+	Mild	98%
A3	27	F	Friedreich's Ataxia	13 years	++	+++	+	Moderate	90%
A4	29	F	Friedreich's Ataxia	10 years	+	+++	+	Mild	99%
A5	34	M	Unknown	1.5 years	+	+++	++	Moderate	96%

– no impairment; + mild impairment; ++ moderate impairment; +++ severe impairment

### Stimuli

Sequence stimuli ranged from one to five syllables and were comprised of consonant-vowel (CV) pairs with the vowel /a/ held constant for each syllable. The complexity of a sequence was determined by the consonants in the sequence and was based on their phonemic similarity and their phonotactic probabilities. Phonemic similarity reflected the number of shared phonetic features (i.e., place of articulation, manner of articulation, and voicing) between consonants in adjacent syllables. In sequences with low phonemic similarity, the consonants of adjacent syllables shared no phonetic features and in sequences with high phonemic similarity these consonants shared two of three phonetic features. Because the sequences in this study were comprised of CV syllables containing the same vowel, they were equally similar to each other at a phonologic level. As a result, the similarity or dissimilarity of a sequence was specific to the phonemic level of the speech sequences. The phonotactic probability of a sequence was either high or low depending on the overall and position-specific probability of a sequence’s syllables and the position-specific phoneme and biphone probability of a sequence’s phonemes. The overall probability of a syllable denoted that syllable’s frequency in English and the position-dependent probability of a syllable or phoneme denoted the frequency that a syllable or phoneme occurred in a particular word position in English. Position-specific biphone probability reflected the frequency of occurrence for two adjacent phonemes in a particular word position. Phonemic similarity and phonotactic probability were combined according to their interfering or facilitating effects on speech reaction times and error rates in previous studies. As a result, *low* complexity sequences possessed low phonemic similarity and high phonotactic probability and *high* complexity sequences contained high phonemic similarity and low phonotactic probability. 

Sequences were balanced across complexity conditions such that each condition contained three different five-syllable combinations that were presented in the same order at one of five different lengths. This resulted in a set of fifteen stimuli for each level of complexity. These stimuli are displayed by complexity in Table 3. The experiment consisted of five runs containing 60 trials and each stimulus was presented on two trials in a run. 

**Table 3:** High and low complexity sequences.

	Utterance Length (in syllables)				
	1	2	3	4	5
Low Complexity	ma	ma ka	ma ka na	ma ka na ha	ma ka na ha da
	da	da ha	da ha na	da ha na ka	da ha na ka ma
	ta	ta ma	ta ma ka	ta ma ka ha	ta ma ka ha na
High Complexity	ja	ja cha	ja cha va	ja cha va za	ja cha va za tha
	va	va za	va za tha	va za tha sha	va za tha sha cha
	tha	tha sha	tha sha za	tha sha za va	tha sha za va cha

### Experimental Procedures

Prior to commencing the experiment, speakers participated in a familiarization procedure that was designed to ensure “whole-word” production of the sequence stimuli during the experiment. The familiarization procedure involved a minimum of 120 practice trials, during which speakers responded to a minimum of two auditory and two visual presentations of each of the sequence stimuli. Extra practice sets were completed in the event of frequent mispronunciations or consistent “segmenting” of the sequence (i.e., not reading the stimulus as a whole word). In addition to ensuring whole-word production of the sequences, the familiarization would have also minimized any effects related to orthographic length (i.e., three of the syllables contained three letters while the remaining syllables all contained two letters) and clarified that the production of the grapheme, ‘th’, was to be unvoiced in the present study. The design of the sequence stimuli in the Spencer and Rogers study was well-suited to detecting differences in serial processing times during production of high and low complexity sequences. Specifically, because sequences were comprised of CV syllables containing the same vowel, changes in the duration of the vowel or the pause time following the vowel could be attributed to differences in the processing time to select the next sequence item and not to any differences in the articulatory durations of phonemes in the sequence. A previous study examined the effects of sequence length and practice on speech error rates and speech durations in this dataset [55] and the present study addressed sequence complexity effects on these variables. 

Speech productions that met any of the following criteria were defined as errors: 1) syllable/sound omission, insertion, transposition, or repetition, 2) initial syllable perseveration of previous target, 3) errors of consonant voicing, manner or place of articulation, 4) syllable segmentation/isolation, 5) self-corrections, and 6) unintelligible responses. Distortions were only coded as an error if they rendered the syllable/word unintelligible or fit the preceding criteria. Twenty percent of the total trials (one randomized block per speaker) were independently scored by a second examiner. Interjudge scoring agreement was 93.8% for the control participants and 90.1% for the clinical participants. Reliability was assessed conservatively using a strict agreement procedure, so that only identical coding of error type(s) was counted as an agreement. The effect of sequence complexity on speech error rates was measured by calculating the proportion of sequences containing at least one error in each complexity condition for each speaker. These values were then arc-sin transformed and multiplied by 100. 

Sequence complexity effects on chronometric measures of speech duration were only evaluated for trials that did not contain errors and were assessed by measuring the duration of the vowel /a/ in each syllable and the pause time, if any, occurring after the vowel but prior to the onset of the next syllable. The latter measurement, termed the *no-consonant interval*, characterized the interval between consecutive syllable onsets independent of the consonant duration in each syllable. It was important to exclude consonant durations as high and low complexity sequences contained different consonants whose different intrinsic durations might obscure the effects of serial processing on durations. 

Differences in the vowel durations and no-consonant intervals were evaluated to investigate the effects of complexity on the selection of individual speech items during sequence production. Increases in these chronometric variables, for example, would indicate an increase in the time to select the next item in a sequence. Because the analysis of vowel durations and no-consonant intervals addressed selection processes, sequence final syllables were excluded from the dataset as production of these syllable would not be influenced by processes to select the next sequence item. One-syllable sequences were excluded for this same reason and, as a result, only twenty-four of the thirty sequences were examined in this study. 

Vowel duration was derived by subtracting each onset for /a/ from the corresponding offset. The no-consonant interval was derived by subtracting the onset for /a/ from the onset of the following syllable. Identification of the speech onsets and offsets for calculating vowel durations and no-consonant intervals was based on time-varying changes in the spectral features associated with the production of vowel /a/ and the manner of articulation of the consonants in a speech sequence. The spectral feature for the vowel /a/ corresponded to the magnitude of spectral energy in the region of F1 (Howitt, 2000) and was derived by digitally bandpass filtering the microphone signal with a 5th order Butterworth filter. The passbands of this filter were speaker-specific and corresponded to the minimum and maximum F1 frequencies produced by a speaker during the first 25 tokens of the first and fifth runs. A dB time series reflecting ‘vowel intensity’ was then derived from the bandpass filtered signal using a 12 ms window that was incremented in 4 ms steps (effective sampling rate = 250 Hz).

The production of nasal consonants is characterized by prominent spectral energy at the lower end of the speech spectrum. Detection of energy associated with nasal consonants was accomplished by digitally bandpass filtering the speech signal between 60 Hz and 500 Hz. A dB time series for ‘nasal intensity’ was then derived using the same parameters that were used for the vowel /a/. Feature detection for stop consonants and affricate consonants was performed by high-pass filtering the microphone signal at 2000 Hz. Because stop and affricate consonants are associated with more abrupt onsets than either vowels or nasals, the dB times series for these consonants was derived from a 6 ms window that was incremented in 2 ms steps (effective sampling rate = 500 Hz). Lastly, energy associated with fricative phonemes was estimated from time series estimates of the 1st spectral moment that were calculated using a 12 ms analysis window incremented in 4 ms steps. 

A smoothed derivative of each of these time series was then calculated to provide information regarding time-varying changes in these spectral features over the course of a speech sequence. The smoothed derivative was calculated by convolving each dB time series with a derivative kernel whose length was adjusted to control for the different onset rates of the sounds in the study. Peaks and valleys in a derivative time series indicated possible onsets and offsets of the corresponding spectral feature. For vowels, the smoothed derivative was derived by convolving the vowel dB time series with a 60 ms 1st order Gaussian derivative kernel. For nasals and fricatives, the length of the derivative Gaussian kernel was 28 ms and for affricates and stops it was 14 ms. The temporal parameters for deriving the dB and derivative time series for different phonemes were determined based on extensive pilot analyses of speech data from both healthy speakers and speakers with dysarthria. For nasal consonants, the ‘onset’ peak occasionally occurred slightly before the offset of the preceding vowel. When this happened, the onset of the nasal consonant was set to the next time point after the vowel offset. 

**Figure 1:**
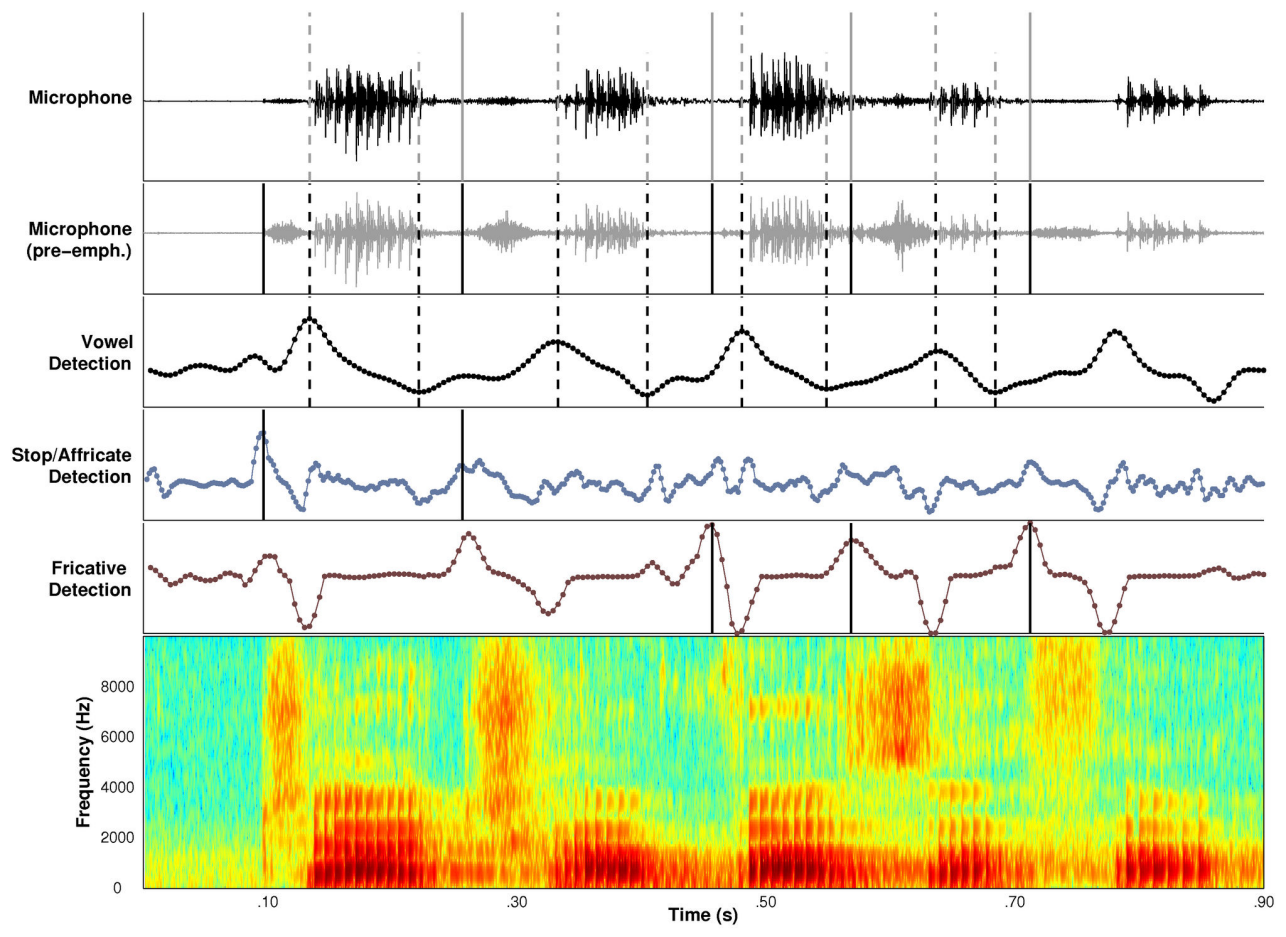
An example of the graphical user interface used to analyze speech sequence productions for each trial. The microphone signal is displayed in the top panel and the pre-emphasized microphone signal in the second panel from the top. The next three panels display time-varying changes in the spectral features of vowels, stops and affricates, and fricatives. The time-varying signals in these panels were used to detect the onsets of the consonants in the sequence and the onsets and offsets of the vowels in the sequence. The bottom panel depicts a broadband spectrogram of the speech sequence produced by the speaker. Gray dashed lines indicate vowel onsets and vowel offsets in the ‘Vowel Detection’ panel. Consonant onsets are indicated by solid black lines in the ‘Detection’ panel describing their manner of articulation. The derived onsets and offsets are shown together in the pre-emphasized microphone panel. The microphone panel demonstrates how the onset and offset values were used to calculate the four vowel durations and no-consonant intervals in the sequence.

A graphical user interface (GUI) was developed to evaluate the outputs of the automated procedures and perform corrections as needed. An example of the GUI’s display for a speaker’s production of the sequence, *ja cha va za tha*, is shown in Figure 1. The ‘Detection’ panels in the figure show the smoothed derivative signals for the different sounds classes in the sequence. Consonant onsets are indicated with solid black lines in the detection panel corresponding to their manner of production. Vowel onsets and offsets are indicated with dashed gray lines in the ‘vowel detection’ panel. The consonant and vowel landmarks are also indicated in the panel displaying the pre-emphasized microphone signal for that trial. The microphone signal displayed in the top panel of Figure 1 shows the four vowel durations and four no-consonant intervals that were derived from these vowel and consonants landmarks. The bottom panel of GUI shows a broadband spectrogram of the speech sequence. The GUI provided playback of the microphone signal as well as a syllable-by-syllable playback with a 500 ms pause occurring after each syllable. A trained user reviewed the signals displayed in the GUI and listened to the sequence audio and manually corrected any inaccurate onset or offset values. After a manual correction, the onsets and offsets were re-plotted and the audio was re-segmented so the user could evaluate the accuracy of the corrections. 

For two of the speakers with ataxic dysarthria, the identification of one or more syllable onsets was not possible for some sequences. On these trials, the production of an obstruent consonant contained two distinct releases/plosions separated by a brief period prior to initiating production of the following vowel. Such productions may have reflected articulatory incoordination, which is commonly observed in ataxic dysarthria. As there was no basis for determining which release constituted the ‘true’ onset of the consonant, it was impossible to calculate an ISI for these syllables and so these trials were excluded from the chronometric analyses. These ‘double-releases’ occurred rapidly and were not detected during playback of the whole sequence, but only during playback of the segmented sequence and an analysis of the spectrogram. For this reason, it was decided that these trials should also be excluded from the analyses of speech errors. These behaviors were observed in two of the speakers with ataxic dysarthria and occurred on approximately 3% of the trials for one speaker and less than 1% of the trials for the other speaker. 

Statistical testing was carried out on the phonotactic measures in the high and low complexity sequences to verify the phonotactic properties. Overall and position-dependent syllable frequency counts per one million words were calculated using the CELEX database [56]. Position-dependent phoneme and biphone probabilities were derived using the phonotactic probability calculator [57] and were expressed in terms of their log (base 10) frequencies relative to the log (base 10) frequencies of all possible words having a phoneme or biphone in that particular position. The summed probability of each phonotactic measure was calculated for each of twenty-four sequences being studied. Differences between the twelve low complexity sequences and the twelve high complexity sequences were evaluated for each phonotactic measure using independent samples t-tests. The findings of this analysis revealed significant differences between low and high complexity for all phonotactic probability measures, t(22) > 5.29, p<.001. Table 4 displays the summed probabilities of each phonotactic measure averaged across low and high complexity sequences. 

**Table 4:** Summed phonotactic probabilities for high and low complexity sequences.

	Complexity	Mean	Mean Difference
Syllable Frequency	Low	910.41	818.86
	High	91.55	
Position - Specific Syllable Frequency	Low	193.98	174.2
	High	19.78	
Position - Specific Phoneme Probability	Low	0.2442	0.1108
	High	0.1334	
Position - Specific Biphoneme Probability	Low	0.0115	0.01
	High	0.0015	

## Results

Speech error rates, vowel durations and no-consonant intervals were extracted from speech sequences to evaluate differences in the accuracy and duration of serial processing during production of low versus high complexity sequences. Linear mixed effects ANOVAs were used to evaluate the main and interaction effects of sequence complexity and group for each of these variables. Because a separate test was performed for each of the three dependent variables, the p-threshold for significance was set to .05/3 = .0167.

**Figure 2:**
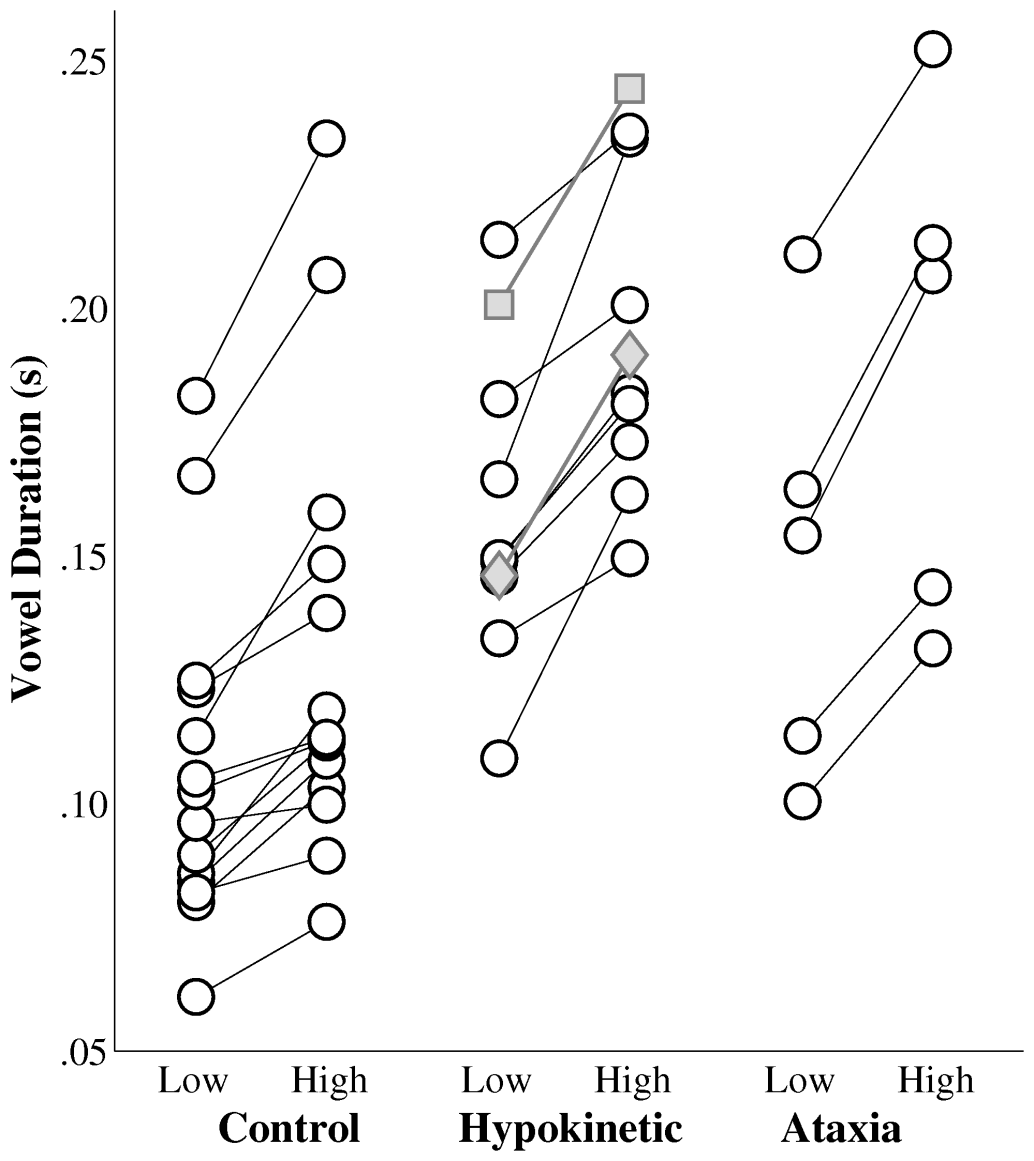
The effects of sequence complexity on speakers’ average vowel durations by group. Data for the speaker with hypokinetic dysarthria from multiple system atrophy is indicated with gray diamonds and for the speaker with hypokinetic dysarthria from corticobasal degeneration with gray squares.

Figure 2 displays speakers’ average vowel durations by complexity and group. A mixed effects ANOVA of speakers’ vowel durations revealed a main effect of group, F(2, 48) = 12.57, p<.001 and comparison of between-group differences in vowel durations was performed using a Bonferroni correction. This comparison revealed that vowel durations in the group of healthy speakers were significantly shorter than those of the speakers with hypokinetic dysarthria (mean difference = 54 ms) and those of the speakers with ataxic dysarthria (mean difference = 50 ms). No significant difference was observed between the vowel durations of the speakers with hypokinetic and ataxic dysarthria. A significant main effect of complexity on speakers’ vowel durations was also observed, F(1, 48) = 8.05, p<.01. Speakers’ vowel durations increased by an average of 33 ms during production of high versus low complexity sequences. No significant interaction was observed between sequence complexity and group, F(2, 48) = 0.23, p=.798. The two speakers with hypokinetic dysarthria secondary to MSA and CBD are displayed in Figure 2 with the data from speakers with hypokinetic dysarthria from Parkinson’s disease. The average vowel durations from the speaker with MSA are indicated with gray diamonds and the average vowel durations from the speaker with CBD are indicated with gray squares. An examination of these data points indicated that the effects of sequence complexity on these speakers’ vowel duration were qualitatively similar to those observed for the speakers with hypokinetic dysarthria from Parkinson’s disease. 

**Figure 3:**
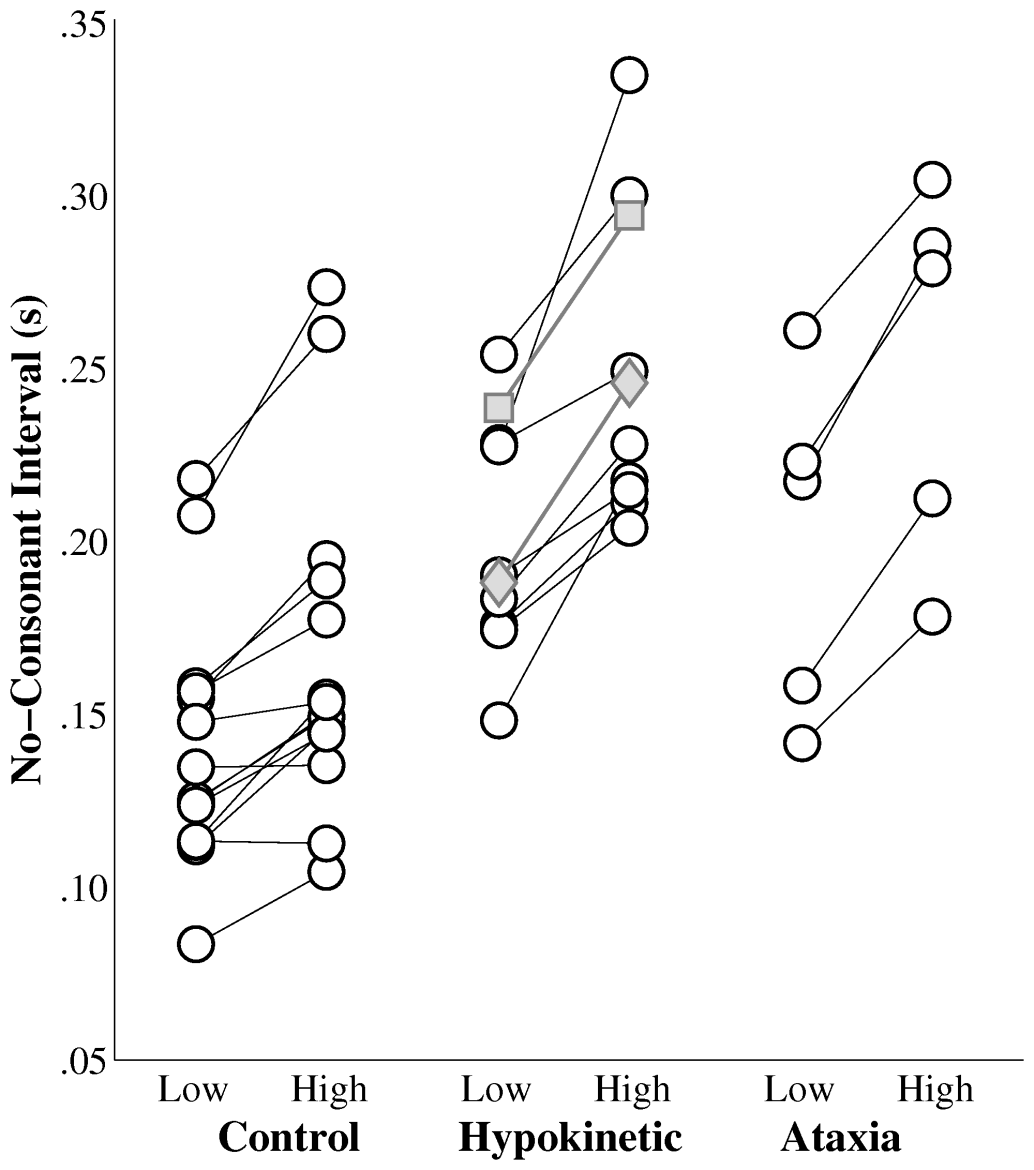
The effects of sequence complexity on speakers’ average no-consonant intervals by group. The gray diamonds indicate the average no-consonant intervals for the speaker with multiple system atrophy and the gray squares indicate the average no-consonant intervals for the speaker with corticobasal degeneration.

Speakers’ average no-consonant intervals are displayed by complexity and group in Figure 3. The results of the mixed ANOVA revealed significant main effects of group and complexity on no-consonant intervals. The main effect for group, F(2, 48) = 16.28, p<.001 showed that healthy speakers producing significantly shorter no-consonant intervals than the speakers with hypokinetic dysarthria (mean difference = 67 ms) and the speakers with ataxic dysarthria (mean difference = 72 ms). No significant difference was observed between the no-consonant intervals produced by the speakers with hypokinetic dysarthria and those produced by the speakers with ataxic dysarthria. In addition, a main effect of sequence complexity was observed, F(1, 48) = 10.05, p<.01, and revealed that no-consonant intervals produced during high complexity sequences were significantly longer than those produced during low complexity sequences by an average of 42 ms. No significant interaction between group and complexity was detected, F(2, 48) = .44, p=.65. As was the case for vowel durations, the mean no-consonant intervals for the speakers with speaker with MSA and the speaker with CBD were qualitatively similar to those of the speakers with Parkinson’s disease. 

In summary, an analysis of speech chronometric variables indicated that production of high complexity sequences was associated with longer vowel durations and longer no-consonant intervals than production of low complexity sequences. The failure to identify an interaction effect between group and either vowel duration or no-consonant intervals indicated that the effects of sequence complexity were similar across speaker groups. 

**Figure 4:**
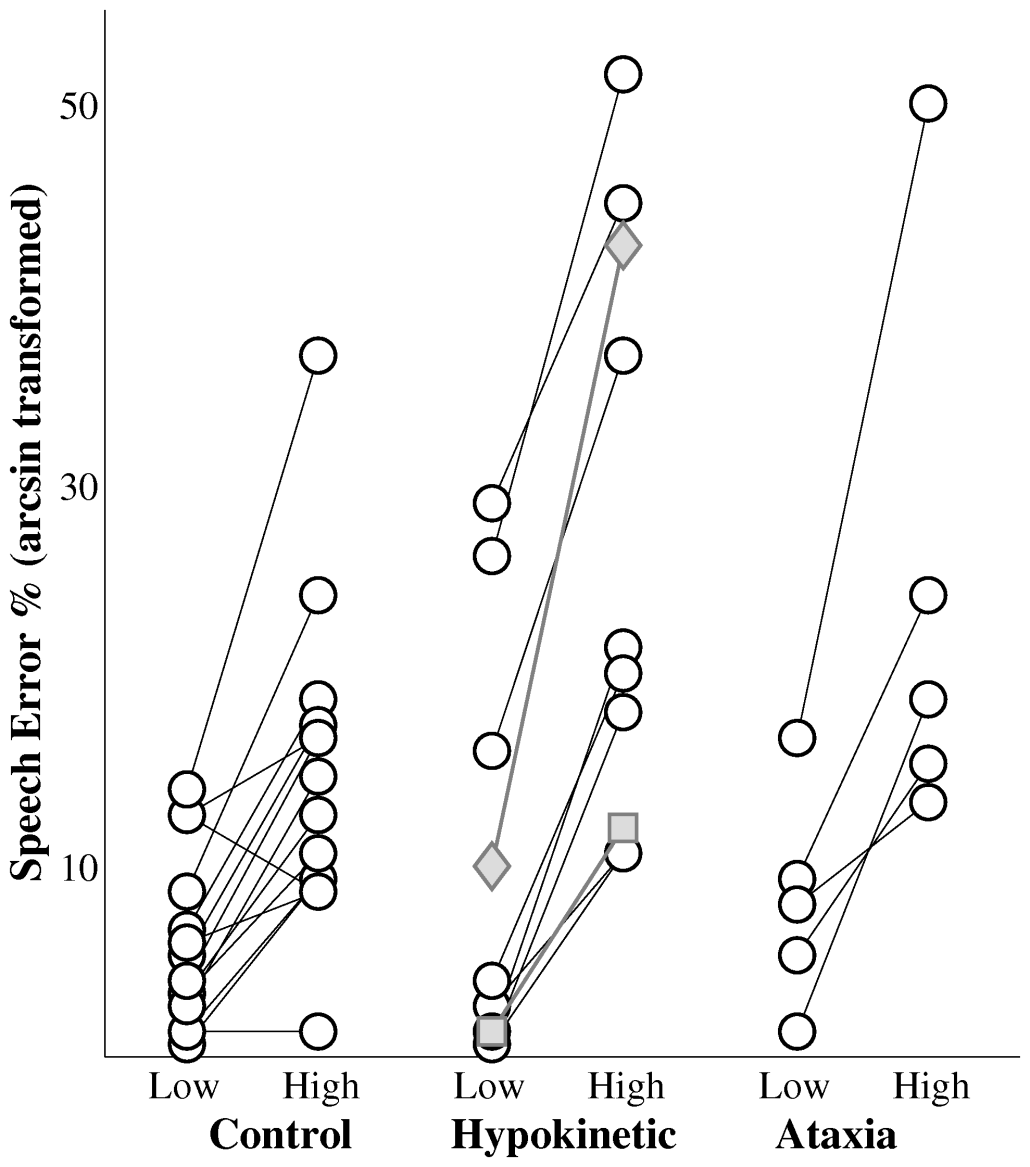
The effects of sequence complexity on speech error rates (arcsin transformed) by group. Data from the speaker with multiple system atrophy (gray diamond) and the speaker with corticobasal degeneration (gray square) are plotted with the data from the speakers with hypokinetic dysarthria from Parkinson’s disease.

The effects of sequence complexity, group and the interaction of sequence complexity and group on speech error rates were also evaluated. This analysis revealed a significant effect of sequence complexity on speech error rates, F(1, 48) = 10.05, p<.01. Error rates during high complexity sequences were significantly higher than those during low complexity sequences by an average of 14.1%. A significant main effect of group was not observed, F(2, 48) = 3.64, p=.034. In addition, no interaction between sequence complexity and group was detected, F(2, 48) = 0.99, p=.38. Figure 4 displays speakers’ error rates by speaker group and by level of complexity. These findings paralleled those of the chronometric analysis in that high complexity sequences elicited significantly higher error rates compared to low complexity sequences and that this effect was similar across speaker groups. For this analysis, sequence complexity effects in the speakers with MSA and CBD were comparable to those of speakers with Parkinson’s disease.

## Discussion

The present study investigated the processes responsible for selecting speech items during speech production and whether the timing and accuracy of these selection processes are affected by sequence complexity. High complexity sequences were associated with higher speech error rates, longer vowel duration and longer no-consonant intervals. These effects were observed for healthy speakers as well as speakers with hypokinetic and ataxic dysarthria. The results of the present study are consistent with findings for speech reaction times and indicate that the phonemic similarity and/or phonotactic probability of sequence items influence serial control for speech production. 

Insights into serial control for speech derive largely from studies of speech error patterns and chronometric variations during different speech tasks. Because both lines of analysis have proved valuable, models of speech serial control must ultimately account for both types of findings. As such, combined analyses of speech errors and chronometric variations provide an effective investigative approach in this effort and have been successful at elaborating even subtle aspects of serial control [9]. In the present study, speech error and chronometric findings (i.e., vowel durations and no-consonant intervals) both indicated that serial control for speech was subject to greater interference during high complexity sequences than during low complexity sequences. While the increased reaction times reported by Spencer and Rogers (Spencer & Rogers, 2005) attest to the effects of sequence complexity on preparatory processing for speech, the current findings indicate that these effects extend throughout sequence production. Together, these findings demonstrate that serial ordering of speech sounds requires more time and is more prone to error when the phonemes in a speech sequence share phonetic features and/or possess lower phonotactic frequencies. In summary, the complexity effects observed during sequence production (i.e., the increased processing times indicated by the longer vowel durations and no-consonant intervals) provides important insight into the ongoing nature of serial processing during sequence production and highlight the value of within-sequence chronometric measures. In addition, the finding that complexity effects during sequence *production* were identical to their effects during sequence *preparation* (i.e., the increased processing times indicated by longer speech reaction times), provide compelling evidence that a portion of serial processing associated with sequence preparation continues to operate during sequence production. 

Although high and low complexity sequences were designed to reflect differences in both phonemic similarity and phonotactic probability, it is possible that the observed complexity effects resulted from only one or the other of these variables. Since phonemic similarity and phonotactic probability have both been associated with longer reaction times and high error rates, reaction time findings of the Spencer and Rogers [53] study and the error rate findings in the present study do not distinguish between similarity and phonotactic effects. Although not studied as extensively as speech reaction times and error rates, a few studies have evaluated the independent effects of phonotactic probability on measures of speech duration. For example, Kendall and colleagues (Kendall et al., 2005) observed an opposite effect of phonotactic probability in their reaction time study of frequent and infrequent inter-phoneme transitions. This study reported that the duration of speakers’ productions of three-syllable non-words was significantly longer when the non-words contained phonemes sequences with low inter-phoneme transition frequencies than when the non-words contained moderate transition frequencies. In addition, the durations of non-words with moderate inter-phoneme transition frequencies were significantly longer than those with low frequency transitions. A similar finding was reported by Bose and colleagues (Bose, van Lieshout, & Square, 2007) for mono-syllabic word durations. The findings of these studies are in contrast to those of Levelt and Wheeldon (Levelt & Wheeldon, 1994) who evaluated the durations of two-syllable words containing syllables with either high or low overall and position-dependent frequencies. These investigators observed significantly shorter word durations for words containing either a high frequency first or second syllable compared to words with either a low frequency first or second syllable. A similar finding was reported by Munson (2001) during production of two syllable CVC non-words. In that study, speech stimuli were constructed such that the biphone frequency of the syllable final consonant and syllable initial consonant separating the two syllables were either high or low. Munson [58] observed that child and adults speakers produced the high frequency biphones with significantly shorter durations than low frequency biphones during both comfortable and rapid speech production rates. In the current study, high and low complexity sequences differed in both syllable frequency and phoneme transition frequencies. The opposite effects of these phonotactic variables on speech durations observed in these investigations make it difficult to assess the contribution of phonotactic probability to the current findings, but indicate that phonotactic probability cannot be ruled out as a contributing factor to the sequence complexity effects observed in the present study. 

Phonemic similarity effects on speech duration measures have not, to our knowledge, been investigated previously and so a comparison with previous findings is not possible. However, similarity-based interference has been addressed in several modelling investigations [10,59–61] and these models provide a framework for considering the phonemic similarity effects observed in the present study. In a class of sequence production models, known as competitive queuing (CQ), similarity-based interference results from the competitive process for selecting the item that is to be produced next in a sequence [10,60,61]. In this competition, an item excites its own activation and inhibits the activations of other items to a degree that is proportional to its current activation level. The item with the largest activation level, or whose activation level exceeds a threshold value, is selected to be the next item in the sequence. In competitive networks such as these, the duration and accuracy of the selection process is largely attributable to the magnitude of noise across item activations and the amount of contrast between the activation levels of competing items. Items that are either phonologically or phonemically similar will have similar activation levels, or less contrast that, in a noisy network, increases the likelihood of selecting the wrong item [10,60,61]. Reduced contrast between activation of similar items will also increase the duration of the competition to select the next sequence item [9]. As a result, the competitive selection process in these models provides a mechanism to account for the higher error rates and longer vowel durations and no-consonant intervals observed during phonemically similar sequences. In addition to the effects of reduced contrast, Rogers and Storkel (Rogers & Storkel, 1998) noted that post-selection inhibition might also contribute to phonemic similarity. That is, if a competitive process mediates the specification of phonetic features in sounds and syllables, inhibition of most recently selected features adversely affects the selection of features for the next item when that item possesses many of the same features. Although CQ-based models are typically applied to phonologic similarity effects, these models could account for the effects of phonemic similarity by parameterizing similarity at a phonemic, instead of phonologic level. Alternatively, parameterizing similarity in terms of speech motor features, as proposed by Grossberg and Pearson [62], could also account for phonemic similarity effects as phonetic features reflect articulatory and acoustic aspects of phoneme production. In summary, the effects of sequence complexity observed in the current study are consistent with the interference effects of phonemic similarity on speech reaction times and error rate effects observed in other studies and are also consistent with computational models that simulate similarity-based interference effects on speech production.

In summary, a review of the findings from previous investigations of phonotactic probability and phonemic similarity does not allow for any definitive conclusions regarding the relative contributions of these factors to the observed effects of sequence complexity in the present study. Nevertheless, a better understanding of the different effects associated with phonemic similarity and phonotactic probability has significant theoretical value for understanding spoken language production. For example, the association between syllable frequency and speech error and chronometric measures has been interpreted as support for the idea that serial control for speech involves the selection of syllable-sized units. In contrast, the interference associated with phonemically similar sequences has been taken as evidence that phonetic features are also represented during the selection process for speech. 

In the present study, speakers with dysarthria differed from the healthy controls in terms of significantly longer vowel durations and no-consonant intervals, with no differences found between the two dysarthria groups. This was also true of the two speakers with hypokinetic dysarthria from MSA and CBD. The speech of individuals with ataxic dysarthria has long been characterized by prolonged phonemes and prolonged intervals [63,64] which are consistent with the often slowed speech rate [40]. These prolongations, and the subsequent effects to rate of speech, have been attributed at least in part to hypotonia [65,66] and increased reliance on auditory feedback during speech [67]. Speakers with hypokinetic dysarthria from Parkinson’s disease, however, have been shown to have vowel durations comparable to healthy speakers [68,69] which is perhaps consistent with the speech rate variability found among individuals with hypokinetic dysarthria [64]. In the present study, where subjects were performing a reaction time task, the longer vowel durations and pauses may be attributed to the reduced rate of speech found on tasks requiring rapid, controlled productions, such as diadochokinetics and syllable repetition tasks [70,71].

There are a paucity of studies related to speech complexity effects in dysarthria, particularly those with ataxic and hypokinetic dysarthria. Contrary to our predictions, the speakers with dysarthria experienced similar sequence complexity effects to the healthy speakers. These findings may indicate that the effects of phonemic similarity and phonotoactic probability are primarily attributable to processing by different regions of cortex. For example, Papoutsi and colleagues [72] analyzed brain activation patterns during production of two- and four-syllable pseudowords containing either high or low phoneme and biphone frequencies. Brain activations associated with the frequency characteristics of pseudowords were observed in left supplementary motor area (SMA), left dorsal pre-central gyrus, and the inferior frontal gyrus bilaterally. Within the inferior frontal activations, a particularly strong association was present for pseudoword frequency activation in the ventral portion of the pars opercularis. The involvement of inferior frontal regions in phonotoactic processing is consistent with findings from other studies [73,74]. For example, Carreiras and colleagues [73] examined changes in brain activity during production of bisyllabic words that varied in word frequency and word-initial syllable frequency. These investigators observed greater activation in the anterior insula during production of words with low versus high initial syllables. Interestingly, the increased insular activity was observed for both high and low frequency words. Riecker and colleagues [74] examined changes in brain activity during production of bisyllabic words that varied in onset complexity (CCV versus CV) and syllable frequency. Although significant activations associated with syllable frequency were not observed, a functional connectivity analysis revealed that the left anterior insular and the posterior portion of the left inferior frontal gyrus exhibited highly correlated hemodynamic response changes during production of low versus high frequency syllables. 

Although definitive findings regarding cortical processing of effects of either phonologic or phonemic similarity are lacking, neural models have posited that processing-related similarity also engages inferior frontal regions of cortex [8,75]. Together these findings attest to the importance of cortical regions, in particular anterior perisylvian regions of cortex, in processing aspects of spoken language such as phonemic similarity and phonotoactic probability and, in particular, to the importance of in anterior perisylvian regions in these processes. 

A companion study investigated sequence length and practice effects in the speakers with dysarthria and revealed sequencing deficits that were specific to the location of damage [55]. Speakers with cerebellar damage exhibited deficits in the capacity to fully plan a sequence prior to production; speakers with Parkinson’s disease exhibited deficits selecting the individual items of a sequence during production. Together, the findings of the companion study and those of the present study suggest that speech sequencing deficits in speakers with either hypokinetic or ataxic dysarthria are specific to particular aspects of serial control. As a result, the finding that complexity effects were not significantly different between the healthy speakers and the speakers with dysarthria may indicate that the distribution of processing related phonemic similarity and phonotoactic probability is not critically dependent on cortical – cerebellar or cortical – basal ganglia loops. Alternatively, the findings of the present study may indicate that cerebellar and basal ganglia processing associated with either phonemic similarity or phonotoactic probability was not adversely affected by deficits exhibited by the present group of speakers with dysarthria. 

The results of the present study confirmed previous findings demonstrating increased error rates during production of sequences with high phonemic similarity and low phonotactic probability. In addition, the increased vowel durations and no-consonant intervals during production of these sequences extend previous reaction time findings and indicate that phonemic similarity and phonotactic probability influence serial processing throughout sequence production. The clinical comparison group of speakers with dysarthria fills a void in the literature on complexity influences in dysarthric speech, and contributes to the integration of normal and disordered speech into a comprehensive model of speech production [76].
